# Asymptomatic SARS-CoV-2 infections: What do we need to know?

**DOI:** 10.1017/ice.2020.201

**Published:** 2020-05-06

**Authors:** Caixia Tan, Yuanyuan Xiao, Xiujuan Meng, Xun Huang, Chunhui Li, Anhua Wu

**Affiliations:** 1 Xiangya Hospital of Central South University, Changsha, Hunan Province, China; 2 National Clinical Research Center for Geriatric Disorders (XiangYa Hospital), Changsha, Hunan Province, China

*To the Editor—*The global outbreak of coronavirus disease 2019 (COVID-19) was officially declared as a pandemic by World Health Organization (WHO) on March 11, 2020,^1^ and it has imposed unprecedented, far-reaching impacts upon public health and the global ecomony. As of April 30, 2020, >3 million cases of COVID-19 have been confirmed, including >210,000 deaths.^2^ Meanwhile, a growing body of are reporting that many COVID-19 infections might present no or only mild symptoms, with a much higher proportion of asymptomatic infections than previously expected.^3–5^ Asymptomatic COVID-19 includes asymptomatic infected persons and presymptomatic infected persons. Those with positive reverse transcription-polymerase chain reaction (RT-PCR) results who never develop any signs or clinically symptoms of COVID-19 are considered asymptomatic infected persons. Those with positive reverse transcription-polymerase chain reaction (RT-PCR) results who fail to show any signs or clinically symptoms of COVID-19 at testing but eventually developed symptoms are considered presymptomatic infected persons. Approximately 60% of COVID-19 cases may have no symptoms or mild symptoms, according to an article published online in *Nature* on March 20.^6^ In fact, as of April 14, 2020, some 6,764 asymptomatic infections have been recognized in mainland China, including 588 imported infections and 1,297 cases that had been recategorized as confirmed cases.^7^ Also, SARS-CoV-2 can not only damage human lungs but can also attack many other organs, including the gut and blood vessels, kidneys, etc, thus presenting different symptoms and signs.^8^ So, why do some infected persons still show no symptoms or only mild symptoms?

The virus is transmitted by exhaled virus-laden droplets that are inhaled by susceptible individuals; these droplets enter the nose and throat, and the virus attacks the cell-surface receptor called angiotensin-converting enzyme 2 (ACE2).^9^ Because SARS-CoV-2 is a new pathogen to this individual, the immune cells do not recognize it and it escapes the defense system of the body and replicates itself to invade new host cells. These host cells are destroyed in this process, and these pathological changes alert the immune system to begin fighting the diseased cells as well as the virus. A recent study indicated that the genes involved in innate immunity are coexpressed in nasal epithelial cells with viral-entry–associated genes.^9^ Thus, if the early immune response can suppress enough viral replication to prevent it from continuing into the lungs, the infected individual could have no or only mild symptoms. Another ex vivo study has shown that SARS-CoV-2 induced significantly less host interferon and proinflammatory response than SARS-CoV, and the low degree of innate immune activation could account for the mild or even lack of symptoms in many COVID-19 patients.^10^ To date, the exact reasons for no or only mild symptoms in many COVID-19 patients remain unclear, and further research is urgently needed to explore the causes and transmission of asymptomatic infections.

The SARS-CoV-2 viral load in upper respiratory specimens is almost as high in asymptomatic infections as symptomatic infections.^11^ Several studies have indicated that asymptomatic and presymptomatic patients can transmit virus to others.^12–14^ A study published in *Nature Medicine* reported that patients with laboratory-confirmed COVID-19 began to shed virus 2–3 days before the onset of symptoms and that their infectivity peaked before symptom onset.^15^ Another study conducted by the Department of Statistics and Actuarial Science of the University of Hong Kong concluded that there was no difference in the transmission rates of coronavirus between symptomatic patients and asymptomatic cases.^16^ Overall, these studies provided evidence that the risk of transmission by asymptomatic patients might be not lower than that by symptomatic patients. Moreover, some individuals infected with the virus experience no or only mild symptoms, and they might be unaware of their disease and thus not isolate themselves or seek treatment. They might be overlooked by healthcare works (HCWs) and possibly trigger a “butterfly effect.” Finally, although many detection methods are available, individuals in the “window period” of COVID-19 infection can be missed, and up to 29% of patients could have an initial RT-PCR false-negative result,^17^ a paper prepublished on the *medRxiv* website suggests, so it is possible that a large portion of asymptomatic infections are going undetected.

All of this evidence indicates that the spread of pandemic of COVID-19 will be difficult to curb by focusing on symptomatic infections alone. Therefore, how can we detect as many asymptomatic infections as possible and hopefully prevent a new wave? First, to achieve universal participation during the pandemic, authorities should fully use mainstream media and the internet to provide timely release of relevant information about the pandemic in an open and responsible manner. Then citizens can correctly understand the severity of the outbreak and act accordingly. Mass media need to disseminate health promotion messages such as indications for wearing a mask and handwashing routine and the importance of maintaining 2-m (6 feet) social distancing. Also, considering that medical supplies are in short supply worldwide, cloth face coverings can be used as an additional, voluntary public health measure.^18^ Third, once asymptomatic infections have been confirmed, self-quarantine is necessary, and these cases should be required to monitor their health status daily, to contact and follow the advice of their medical provider, and to stay home or wear a mask and remain 2 m away from other people if they go out.^19^ Because the rate of asymptomatic SARS-CoV-2 infection may be high among the close contacts of a symptomatic patients, these contacts should be closely monitored to rule out infection, even if they remain asymptomatic. Finally, scientists and public health experts should conduct research on SARS-CoV-2 to quickly improve the detection capacity and to achieve mass testing of citizens, especially those living in large enclosed facilities and those living and working in high-risk facilities (eg, healthcare workers).
